# Re-Vaccination

**Published:** 1902-02-01

**Authors:** Arthur Maude


### Hospital Clinics and Medical Progress.

### RE-VACCINATION.

By Arthur Maude.

The systematic practice of re-vaccination in England is so little followed, the space devoted to the subject in systemic literature is so small, and the points of doubt in the minds of practitioners are obviously so many that even a slight review of the subject may at the present moment be opportune. The average practitioner has no opportunity for any wide grasp of the subject of re-vaccination, for re-vaccination is in England as much limited to a class as the practice of dressing for dinner. To be re-vaccinated is a badge of education and social position. In considering the necessity of re-vaccination we are bound to bear in mind the condition of the population as to primary vaccination. Since the working of the present Act the proportion of children vaccinated publicly has risen considerably, and the proportion who escape vaccination altogether has fallen, but when face to face with an epidemic of small-pox we are concerned with the primary vaccination of many years ago. In 1894 the returns published for the births registered in 1892 show that only 43·8 per cent. of those babies were vaccinated by the public vaccinators, and only 74·5 were vaccinated at all. Of the unvaccinated babies of 1892 probably not a high proportion are still alive. The fact that so many as 25·5 per cent. escaped vaccination altogether is a small matter compared with the question ; what is the quality of the vaccination certified to have been performed ? We may take the high quality of the vaccination performed at the public expense for granted. Parliament defines successful vaccination for them, and the Local Government inspects the work, paying premiums for a high standard of performance. But what of the vaccination undertaken by private practitioners ? The Vaccination Acts enforce no standard of vaccination on them ; instead of making four insertions of lymph, and making a return to the Local Government Board of the result of every insertion of lymph, as the public vaccinator must do, the private practitioner may certify as efficiently vaccinated a child vaccinated in only two, or even one place. There are no official statistics on this head, but the observations recently made in London Board Schools, on children under 13 years old, show that half the children inspected were thus inefficiently vaccinated. Moreover, this evil is not one of quite recent years, for the writer has lately noted the marks on a large number of domestic servants between 20 and 30 years of age, and found fully half presented only one or two scars. The discretion left to the medical profession has unquestionably been abused, and the abuse has even been openly defended in recent medical journals by practitioners who have lent themselves to it.

My purpose is rather to discuss the period of life and other conditions at which re-vaccination should be effected, and the phenomena attending the performance as contrasted with primary vaccination.

A single first-class vaccination in infancy will *modify* small-pox for a lifetime, but it will not protect against infection for more than a term of years, probably from seven to ten. The Commission in its report urges the important and imperative necessity for re-vaccination. They reported that particular classes of officials, such as postmen, policemen, and nurses, who are re-vaccinated systematically are especially protected as compared with persons unre-vaccinated. The figures afforded in Warrington (1892-93) should be sufficient for any mind : of the civil population, about 60,000, there were 17,000 re-vaccinated, and not one person of these was attacked ; of the garrison, 800 strong, only one militiaman escaped re-vaccination, and he was the only case of small-pox.

It has been claimed and, I think, rightly, that every doctor at least should be " absolutely " vaccinated as recommended by Warlomont. This condition is produced by re-vaccination again and again as soon as the effect of one vaccination has disappeared. Recently the writer inquired of his fellow-guests at a large medical dinner how many had been " absolutely vaccinated ; " very few had even heard the term—none had practised it. The writer's personal experience is thus : he was supposed to have been vaccinated in infancy, was re-vaccinated at eight years old with pronounced results, re-vaccinated at 17, and again at 24 with marked result. In 1892 someone had proposed the theory that vaccination was a prophylactic against influenza ; determined to carry this to its logical conclusion the writer re-vaccinated himself four times at intervals of a month with decreasing results, eventually with no reaction ; since then he has regularly vaccinated himself every few months with absolutely no effect, though the lymph has been in all cases above suspicion.

This, I think, is the way for a doctor to be vaccinated, and after two applications it causes no inconvenience at all. So high an authority as Seaton, however, entirely repudiates the necessity, or advisability, of such frequent repetitions, even in the face of a small-pox epidemic. " One thoroughly good primary vaccination to start with, and one careful re-vaccination after puberty, so conducted as to give evidence that the lymph was absorbed, are all that is necessary for the complete protection of the population against small-pox." Seaton further points out the drawbacks of hurried vaccination at a time of panic. The value of re-vaccination may then be discredited by imperfect operations or inferior lymph ; and we have had abundant reason to learn that much of the lymph sold recently in London has been very inefficient, owing to the enormous demands made upon its output.

The inferior potency of glycinerated, stored calf lymph is difficult of explanation ; it is not due to excessive dilution with glycerine, for the proportion of glycerine which should be added to the lymph pulp is now well ascertained, and, I believe, adhered to, by the manufacturers. The manufacturers are not, however, careful in all cases to test their lymph by primary vaccinations on babies. A lymph which will produce a typical vaccine vesicle on a calf will not always produce the same reaction in the human subject, and if from such a lymph " cultivations " are made on a succession of calves, it becomes so attenuated as to be perfectly useless for producing human vaccinia.

*(To be continued.)*

